# Dimethyl 2-[24-acetyl-28-oxo-8,11,14-trioxa-24,27-diaza­penta­cyclo­[19.5.1.1^22,26^.0^2,7^.0^15,20^]octa­cosa-2,4,6,15(20),16,18-hexaen-27-yl]but-2-enedioate

**DOI:** 10.1107/S1600536812030644

**Published:** 2012-07-10

**Authors:** Truong Hong Hieu, Le Tuan Anh, Anatoly T. Soldatenkov, Nadezhda M. Kolyadina, Victor N. Khrustalev

**Affiliations:** a Department of Chemistry, Vietnam National University, 144 Xuan Thuy, Cau Giay, Hanoi, Vietnam; b Organic Chemistry Department, Russian People’s Friendship University, Miklukho-Maklaya Street 6, Moscow, 117198, Russian Federation; c X-ray Structural Centre, A. N. Nesmeyanov Institute of Organoelement Compounds, Russian Academy of Sciences, 28 Vavilov Street, B-334, Moscow 119991, Russian Federation

## Abstract

The title compound, C_31_H_34_N_2_O_9_, is a product of the Michael addition of the cyclic secondary amine subunit of the (bis­pidino)aza-14-crown-4 ether to dimethyl acetyl­ene­dicarboxyl­ate. The mol­ecule comprises a tricyclic system containing the aza-14-crown-3 ether macrocycle and two six-membered piperidinone rings. The aza-14-crown-3-ether ring adopts a bowl conformation with a dihedral angle between the planes of the fused benzene rings of 51.14 (5)°. The central piperidone ring has a boat conformation, whereas the terminal piperidone ring adopts a chair conformation. The dimethyl ethyl­enedicarboxyl­ate fragment has a *cis* configuration with a dihedral angle of 56.56 (7)° between the two carboxyl­ate groups. The crystal packing is stabilized by weak C—H⋯O hydrogen bonds.

## Related literature

For general background, see: Hiraoka (1982 ▶); Pedersen (1988 ▶); Schwan & Warkentin (1988 ▶); Gokel & Murillo (1996 ▶); Bradshaw & Izatt (1997 ▶). For related compounds, see: Levov *et al.* (2006 ▶, 2008 ▶); Komarova *et al.* (2008 ▶); Anh *et al.* (2008 ▶); Anh, Hieu, Soldatenkov, Kolyadina & Khrustalev (2012*a* ▶,*b* ▶); Anh, Hieu, Soldatenkov, Soldatova & Khrustalev (2012 ▶); Hieu *et al.* (2011 ▶); Khieu *et al.* (2011 ▶); Sokol *et al.* (2011 ▶).
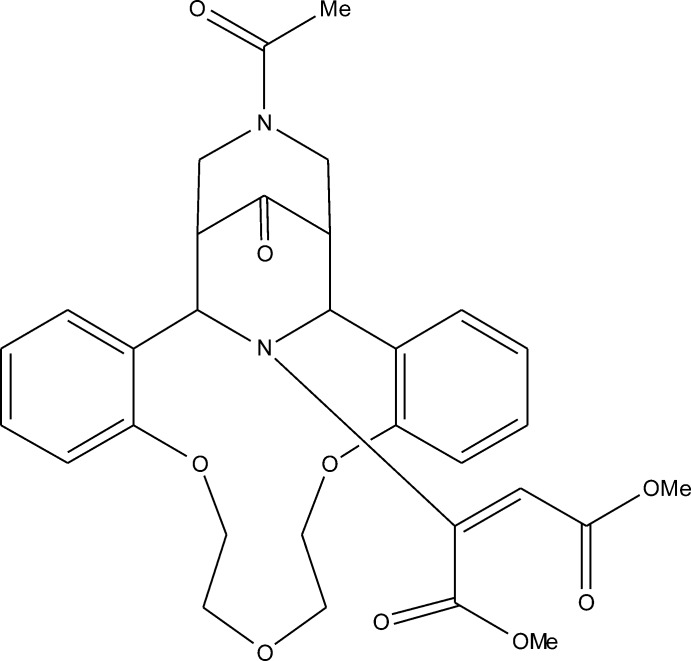


## Experimental

### 

#### Crystal data

C_31_H_34_N_2_O_9_
*M* *_r_* = 578.60
Monoclinic, 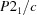

*a* = 9.6634 (6) Å
*b* = 26.3883 (18) Å
*c* = 11.4375 (8) Å
β = 99.614 (1)°
*V* = 2875.6 (3) Å^3^
*Z* = 4
Mo *K*α radiation
μ = 0.10 mm^−1^
*T* = 100 K
0.30 × 0.20 × 0.20 mm

#### Data collection

Bruker APEXII CCD diffractometer
Absorption correction: multi-scan (*SADABS*; Sheldrick, 2003 ▶) *T*_min_ = 0.971, *T*_max_ = 0.981
36500 measured reflections
8396 independent reflections
6209 reflections with *I* > 2σ(*I*)
*R*_int_ = 0.045

#### Refinement

*R*[*F* ^2^ > 2σ(*F* ^2^)] = 0.045
*wR*(*F* ^2^) = 0.111
*S* = 1.00
8396 reflections
382 parameters
H-atom parameters constrained
Δρ_max_ = 0.40 e Å^−3^
Δρ_min_ = −0.26 e Å^−3^

### 

Data collection: *APEX2* (Bruker, 2005 ▶); cell refinement: *SAINT* (Bruker, 2001 ▶); data reduction: *SAINT*; program(s) used to solve structure: *SHELXTL* (Sheldrick, 2008 ▶); program(s) used to refine structure: *SHELXTL*; molecular graphics: *SHELXTL*; software used to prepare material for publication: *SHELXTL*.

## Supplementary Material

Crystal structure: contains datablock(s) global, I. DOI: 10.1107/S1600536812030644/rk2370sup1.cif

Structure factors: contains datablock(s) I. DOI: 10.1107/S1600536812030644/rk2370Isup2.hkl

Supplementary material file. DOI: 10.1107/S1600536812030644/rk2370Isup3.cml

Additional supplementary materials:  crystallographic information; 3D view; checkCIF report

## Figures and Tables

**Table 1:** Hydrogen-bond geometry (Å, °)

*D*—H⋯*A*	*D*—H	H⋯*A*	*D*⋯*A*	*D*—H⋯*A*
C18—H18⋯O35^i^	0.95	2.47	3.1735 (17)	131
C25—H25*A*⋯O33^ii^	0.99	2.30	3.2091 (17)	152
C34—H34*A*⋯O35^iii^	0.98	2.53	3.5045 (19)	174

Symmetry codes: (i) 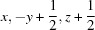; (ii) 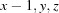; (iii) 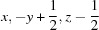.

**Fig. 1.**
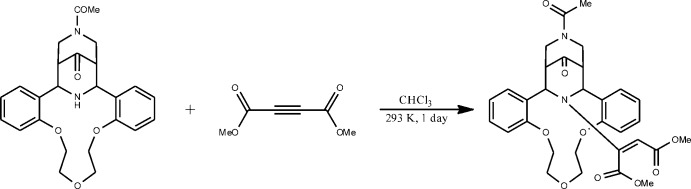
Michael addition of bis(benzo)-(bispidino)aza-14-crown-4 ether to dimethyl acetylenedicarboxylate.

**Fig. 2.**
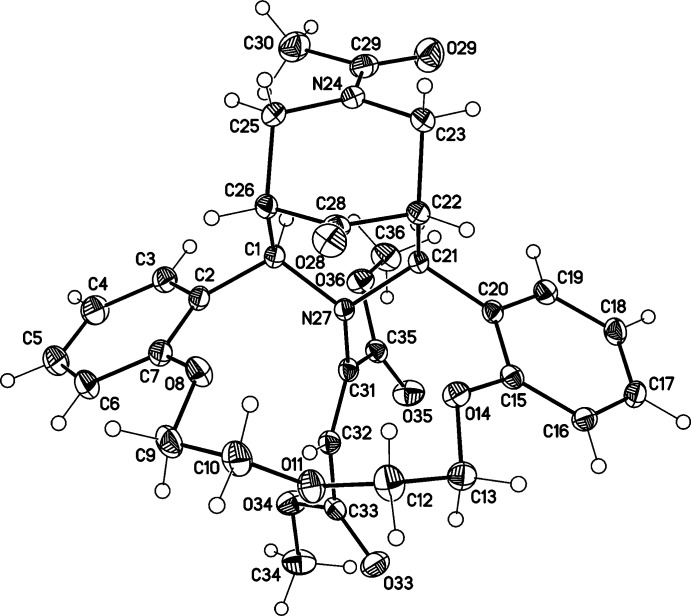
Molecular structure of I with the atom numbering scheme. Displacement ellipsoids are shown at the 50% probability level. H atoms are presented as small spheres of arbitrary radius.

## supplementary crystallographic information

### Comment

Azacrown ethers draw very great attention of investigators over the last half century owing to their great potential for both theoretical and practical interest (Hiraoka, 1982; Pedersen, 1988; Gokel & Murillo, 1996; Bradshaw & Izatt, 1997). Recently we have designed one more effective route to reach this fascinating region of macroheterocyclic compounds, namely, the effective method of synthesis of azacrown ethers containing piperidine (Levov *et al.*, 2006, 2008; Anh *et al.*, 2008; Anh, Hieu, Soldatenkov, Kolyadina & Khrustalev, 2012*a*; Anh, Hieu, Soldatenkov, Soldatova & Khrustalev, 2012), perhydropyrimidine (Hieu *et al.*, 2011), perhydrotriazine (Khieu *et al.*, 2011) and bispidine (Komarova *et al.*, 2008; Sokol *et al.*, 2011; Anh, Hieu, Soldatenkov, Kolyadina & Khrustalev, 2012*b*) subunits.

In attempts to develop the chemistry for new azacrown systems and to obtain macrocyclic ligands bringing the desirable functional groups, we studied the Michael addition of the cyclic secondary amine subunit of the (bispidino)aza-14-crown-4 ether to dimethyl acetylenedicarboxylate. The expected reaction is well known (Schwan & Warkentin, 1988), but might be highly hindered in the case of (bispidino)azacrown system due to the steric reasons. We have found that the expected *N*-vynilation reaction of the (bispidino)azacrown ether proceeded smoothly to give an *N*-maleinate derivative of the azacrown system with a good yield (Fig. 1).

The molecule of I, C_31_H_34_N_2_O_9_, comprises a tricyclic system containing the aza-14-crown-3-ether macrocycle and two six-membered piperidinone rings (Fig. 2). The aza-14-crown-3-ether ring adopts a bowl conformation. The configuration of the C7–O8–C9–C10–O11–C12–C13–O14–C15 polyether chain is *t*-*g(-)*-*t*-*t*-*g*(+)-*t* (*t* = *trans*, 180°; *g* = *gauche*, ±60°). The dihedral angle between the planes of the benzene rings fused to the aza-14-crown-4-ether moiety is 51.14 (5)°. The central piperidone ring has a boat conformation, whereas the terminal piperidone ring adopts a chair conformation. The nitrogen N24 atom has a trigonal-planar geometry (sum of the bond angles is 360.0°), while the nitrogen N27 atom adopts a trigonal-pyramidal geometry (sum of the bond angles is 340.5°). The dimethyl ethylenedicarboxylate fragment has a *cis* configuration with a dihedral angle of 56.56 (7)° between the two carboxylate groups.

The molecule of I possesses four asymmetric centers at the C1, C21, C22 and C26 carbon atoms and can have potentially numerous diastereomers. The crystal of I is racemic and consists of enantiomeric pairs with the following relative configuration of the centers: *rac*-1*R**, 21*S**,22*R**,26*S**.

In the crystal, the molecules of I are bound by the weak intermolecular C–H···O hydrogen bonding interactions into three-dimensional framework (Table 1).

### Experimental

Dimethylacetylenedicarboxylate (0.24 g, 1.69 mmol) was added to a solution of (bispidino)aza-14-crown-4ether (0.25 g, 0.57 mmol) in chloroform (20 ml). The reaction mixture was stirred at 293 K for one day (monitoring by *TLC* until disappearance of the starting organic compounds spots). At the end of the reaction, the formed precipitate was separated, washed with cold chloroform (15 ml) and re-crystallized from ethanol to give 0.32 g of colourless crystals of I. Yield is 98%. *M*.p. = 522-524 K. IR (KBr), ν/cm^-1^: 1603, 1651, 1715. ^1^H NMR (CDCl_3_, 400 MHz, 300 K): δ = 2.33 (s, 3H, CH_3_C═O), 3.02 (m, 2H, H22 and H26), 3.28 and 3.43 (both s, 3H each, OCH_3_), 3.79-4.10 (m, 12H, OCH_2_CH_2_OCH_2_CH_2_O, 2H23 and 2H25), 4.4 and 4.56 (both d, 1H each, H1 and H21, *J* = 7.3), 6.56 (s, 1H, C═CHCOO), 6.70-6.78 (m, 4H, H_arom_), 7.05 (d, 2H, H3 and H19, *J* = 7.6), 7.21 (m, 2H, H_arom_). Anal. Calcd for C_31_H_34_ N_2_O_9_: C, 64.35; H, 5.92; N, 4.84. Found: C, 64.41; H, 6.07; N, 4.67.

### Refinement

The hydrogen atoms were placed in calculated positions with C–H = 0.95-1.00Å and refined in the riding model with fixed isotropic displacement parameters: *U*_iso_(H) = 1.5*U*_eq_(C) for the methyl group and 1.2*U*_eq_(C) for the other groups.

### Crystal data

**Table:**

C_31_H_34_N_2_O_9_	*F*(000) = 1224
*M**_r_* = 578.60	*D*_x_ = 1.337 Mg m^−^^3^
Monoclinic, *P*2_1_/*c*	Melting point = 522–524 K
Hall symbol: -P 2ybc	Mo *K*α radiation, λ = 0.71073 Å
*a* = 9.6634 (6) Å	Cell parameters from 6686 reflections
*b* = 26.3883 (18) Å	θ = 2.3–30.4°
*c* = 11.4375 (8) Å	µ = 0.10 mm^−^^1^
β = 99.614 (1)°	*T* = 100 K
*V* = 2875.6 (3) Å^3^	Prism, light yellow
*Z* = 4	0.30 × 0.20 × 0.20 mm

### Data collection

**Table:**

Bruker APEXII CCD diffractometer	8396 independent reflections
Radiation source: fine-focus sealed tube	6209 reflections with *I* > 2σ(*I*)
Graphite monochromator	*R*_int_ = 0.045
φ and ω scans	θ_max_ = 30.0°, θ_min_ = 1.5°
Absorption correction: multi-scan (*SADABS*; Sheldrick, 2003)	*h* = −13→13
*T*_min_ = 0.971, *T*_max_ = 0.981	*k* = −37→36
36500 measured reflections	*l* = −16→16

### Refinement

**Table:**

Refinement on *F*^2^	Primary atom site location: structure-invariant direct methods
Least-squares matrix: full	Secondary atom site location: difference Fourier map
*R*[*F*^2^ > 2σ(*F*^2^)] = 0.045	Hydrogen site location: inferred from neighbouring sites
*wR*(*F*^2^) = 0.111	H-atom parameters constrained
*S* = 1.00	*w* = 1/[σ^2^(*F*_o_^2^) + (0.046*P*)^2^ + 1.09*P*] where *P* = (*F*_o_^2^ + 2*F*_c_^2^)/3
8396 reflections	(Δ/σ)_max_ < 0.001
382 parameters	Δρ_max_ = 0.40 e Å^−^^3^
0 restraints	Δρ_min_ = −0.26 e Å^−^^3^

### Special details

**Table:**

Geometry. All s.u.'s (except the s.u. in the dihedral angle between two l.s. planes) are estimated using the full covariance matrix. The cell s.u.'s are taken into account individually in the estimation of s.u.'s in distances, angles and torsion angles; correlations between s.u.'s in cell parameters are only used when they are defined by crystal symmetry. An approximate (isotropic) treatment of cell s.u.'s is used for estimating s.u.'s involving l.s. planes.
Refinement. Refinement of *F*^2^ against ALL reflections. The weighted *R*-factor *wR* and goodness of fit *S* are based on *F*^2^, conventional *R*-factors *R* are based on *F*, with *F* set to zero for negative *F*^2^. The threshold expression of *F*^2^ > σ(*F*^2^) is used only for calculating *R*-factors(gt) *etc*. and is not relevant to the choice of reflections for refinement. *R*-factors based on *F*^2^ are statistically about twice as large as those based on *F*, and *R*-factors based on ALL data will be even larger.

### Fractional atomic coordinates and isotropic or equivalent isotropic displacement parameters (Å^2^)

**Table:**

	*x*	*y*	*z*	*U*_iso_*/*U*_eq_	
C1	0.11569 (13)	0.11896 (5)	0.20020 (11)	0.0157 (2)	
H1	0.0768	0.1525	0.2195	0.019*	
C2	0.09245 (13)	0.11385 (5)	0.06665 (11)	0.0179 (3)	
C3	0.02840 (14)	0.15322 (6)	−0.00310 (12)	0.0216 (3)	
H3	0.0081	0.1840	0.0335	0.026*	
C4	−0.00658 (16)	0.14824 (6)	−0.12602 (13)	0.0276 (3)	
H4	−0.0504	0.1754	−0.1727	0.033*	
C5	0.02316 (16)	0.10347 (6)	−0.17898 (13)	0.0287 (3)	
H5	−0.0025	0.0996	−0.2624	0.034*	
C6	0.08995 (15)	0.06399 (6)	−0.11215 (12)	0.0250 (3)	
H6	0.1117	0.0336	−0.1497	0.030*	
C7	0.12495 (14)	0.06917 (5)	0.01056 (12)	0.0200 (3)	
O8	0.18727 (11)	0.03213 (4)	0.08372 (8)	0.0225 (2)	
C9	0.24451 (16)	−0.01130 (6)	0.03346 (13)	0.0250 (3)	
H9A	0.3061	−0.0007	−0.0232	0.030*	
H9B	0.1685	−0.0329	−0.0088	0.030*	
C10	0.32707 (16)	−0.03936 (5)	0.13614 (13)	0.0250 (3)	
H10A	0.2677	−0.0460	0.1972	0.030*	
H10B	0.3595	−0.0723	0.1092	0.030*	
O11	0.44422 (10)	−0.00887 (4)	0.18432 (8)	0.0223 (2)	
C12	0.50802 (16)	−0.02495 (5)	0.29917 (13)	0.0250 (3)	
H12A	0.5739	−0.0531	0.2926	0.030*	
H12B	0.4353	−0.0373	0.3438	0.030*	
C13	0.58570 (15)	0.01879 (5)	0.36341 (13)	0.0220 (3)	
H13A	0.6447	0.0070	0.4376	0.026*	
H13B	0.6472	0.0350	0.3131	0.026*	
O14	0.48284 (9)	0.05405 (3)	0.38952 (8)	0.01810 (19)	
C15	0.52964 (13)	0.09761 (5)	0.44734 (11)	0.0154 (2)	
C16	0.66552 (13)	0.10392 (5)	0.50956 (11)	0.0175 (3)	
H16	0.7331	0.0778	0.5094	0.021*	
C17	0.70186 (14)	0.14851 (5)	0.57176 (12)	0.0202 (3)	
H17	0.7942	0.1525	0.6148	0.024*	
C18	0.60535 (14)	0.18708 (5)	0.57162 (12)	0.0202 (3)	
H18	0.6306	0.2175	0.6143	0.024*	
C19	0.47031 (14)	0.18075 (5)	0.50794 (11)	0.0182 (3)	
H19	0.4042	0.2075	0.5071	0.022*	
C20	0.42963 (13)	0.13655 (5)	0.44570 (11)	0.0147 (2)	
C21	0.27882 (13)	0.13019 (5)	0.38529 (11)	0.0144 (2)	
H21	0.2286	0.1629	0.3921	0.017*	
C22	0.20355 (13)	0.08817 (5)	0.44644 (11)	0.0158 (2)	
H22	0.2728	0.0704	0.5076	0.019*	
C23	0.08405 (14)	0.11077 (5)	0.50463 (11)	0.0191 (3)	
H23A	0.0399	0.0836	0.5455	0.023*	
H23B	0.1229	0.1364	0.5644	0.023*	
N24	−0.02109 (11)	0.13426 (5)	0.41473 (10)	0.0192 (2)	
C25	−0.08063 (13)	0.10019 (6)	0.31856 (12)	0.0207 (3)	
H25A	−0.1472	0.1192	0.2591	0.025*	
H25B	−0.1331	0.0727	0.3506	0.025*	
C26	0.03642 (13)	0.07711 (5)	0.25846 (11)	0.0168 (2)	
H26	−0.0050	0.0520	0.1970	0.020*	
N27	0.26543 (11)	0.11722 (4)	0.25751 (9)	0.0146 (2)	
C28	0.13448 (13)	0.05052 (5)	0.35589 (11)	0.0173 (3)	
O28	0.14211 (11)	0.00491 (4)	0.36862 (9)	0.0246 (2)	
C29	−0.05451 (14)	0.18412 (6)	0.42329 (13)	0.0233 (3)	
O29	0.00129 (12)	0.21020 (4)	0.50702 (10)	0.0323 (3)	
C30	−0.16326 (16)	0.20626 (6)	0.32678 (15)	0.0304 (3)	
H30A	−0.1776	0.2421	0.3437	0.046*	
H30B	−0.2519	0.1879	0.3236	0.046*	
H30C	−0.1309	0.2032	0.2503	0.046*	
C31	0.36237 (13)	0.14313 (5)	0.19759 (11)	0.0149 (2)	
C32	0.44149 (13)	0.11639 (5)	0.13532 (11)	0.0162 (2)	
H32	0.4245	0.0809	0.1296	0.019*	
C33	0.55331 (14)	0.13640 (5)	0.07407 (11)	0.0170 (2)	
O33	0.67355 (10)	0.12234 (4)	0.09900 (9)	0.0259 (2)	
O34	0.50805 (10)	0.16863 (4)	−0.01385 (9)	0.0238 (2)	
C34	0.61758 (16)	0.18984 (6)	−0.07172 (14)	0.0277 (3)	
H34A	0.5771	0.2155	−0.1293	0.042*	
H34B	0.6600	0.1628	−0.1127	0.042*	
H34C	0.6896	0.2055	−0.0122	0.042*	
C35	0.37909 (14)	0.19960 (5)	0.21065 (11)	0.0172 (2)	
O35	0.48639 (11)	0.22224 (4)	0.20779 (10)	0.0258 (2)	
O36	0.25872 (10)	0.22096 (4)	0.22817 (9)	0.0209 (2)	
C36	0.25952 (17)	0.27511 (5)	0.24882 (14)	0.0266 (3)	
H36A	0.1657	0.2889	0.2206	0.040*	
H36B	0.3274	0.2913	0.2059	0.040*	
H36C	0.2859	0.2818	0.3339	0.040*	

### Atomic displacement parameters (Å^2^)

**Table:**

	*U*^11^	*U*^22^	*U*^33^	*U*^12^	*U*^13^	*U*^23^
C1	0.0137 (6)	0.0194 (6)	0.0137 (6)	−0.0016 (5)	0.0010 (4)	−0.0006 (5)
C2	0.0145 (6)	0.0247 (7)	0.0141 (6)	−0.0040 (5)	0.0011 (4)	−0.0005 (5)
C3	0.0203 (6)	0.0263 (7)	0.0179 (6)	−0.0004 (5)	0.0025 (5)	0.0014 (5)
C4	0.0263 (7)	0.0376 (9)	0.0184 (7)	0.0024 (6)	0.0023 (6)	0.0072 (6)
C5	0.0285 (7)	0.0429 (9)	0.0142 (6)	−0.0008 (7)	0.0026 (5)	0.0000 (6)
C6	0.0265 (7)	0.0325 (8)	0.0160 (6)	−0.0032 (6)	0.0039 (5)	−0.0050 (6)
C7	0.0178 (6)	0.0250 (7)	0.0171 (6)	−0.0034 (5)	0.0023 (5)	−0.0011 (5)
O8	0.0290 (5)	0.0207 (5)	0.0175 (5)	0.0017 (4)	0.0031 (4)	−0.0036 (4)
C9	0.0301 (7)	0.0218 (7)	0.0223 (7)	−0.0007 (6)	0.0019 (6)	−0.0092 (6)
C10	0.0309 (7)	0.0165 (7)	0.0270 (7)	−0.0033 (6)	0.0027 (6)	−0.0063 (5)
O11	0.0263 (5)	0.0194 (5)	0.0201 (5)	−0.0024 (4)	0.0011 (4)	−0.0001 (4)
C12	0.0335 (8)	0.0162 (7)	0.0237 (7)	0.0036 (6)	−0.0001 (6)	−0.0005 (5)
C13	0.0219 (6)	0.0188 (7)	0.0245 (7)	0.0055 (5)	0.0012 (5)	−0.0030 (5)
O14	0.0171 (4)	0.0143 (4)	0.0229 (5)	−0.0004 (3)	0.0035 (4)	−0.0024 (4)
C15	0.0180 (6)	0.0154 (6)	0.0132 (6)	−0.0016 (5)	0.0039 (5)	0.0008 (5)
C16	0.0163 (6)	0.0194 (6)	0.0171 (6)	0.0021 (5)	0.0037 (5)	0.0020 (5)
C17	0.0158 (6)	0.0269 (7)	0.0168 (6)	−0.0025 (5)	−0.0003 (5)	−0.0002 (5)
C18	0.0215 (6)	0.0210 (7)	0.0172 (6)	−0.0032 (5)	0.0006 (5)	−0.0045 (5)
C19	0.0204 (6)	0.0176 (6)	0.0166 (6)	0.0010 (5)	0.0029 (5)	−0.0008 (5)
C20	0.0157 (6)	0.0165 (6)	0.0117 (5)	−0.0003 (5)	0.0020 (4)	0.0018 (5)
C21	0.0145 (5)	0.0168 (6)	0.0116 (5)	−0.0002 (4)	0.0015 (4)	−0.0003 (4)
C22	0.0156 (6)	0.0185 (6)	0.0135 (6)	−0.0008 (5)	0.0032 (4)	0.0010 (5)
C23	0.0178 (6)	0.0250 (7)	0.0148 (6)	−0.0015 (5)	0.0041 (5)	−0.0014 (5)
N24	0.0140 (5)	0.0260 (6)	0.0178 (5)	−0.0004 (4)	0.0031 (4)	−0.0040 (5)
C25	0.0143 (6)	0.0285 (7)	0.0195 (6)	−0.0041 (5)	0.0034 (5)	−0.0038 (5)
C26	0.0158 (6)	0.0196 (6)	0.0150 (6)	−0.0041 (5)	0.0028 (5)	−0.0025 (5)
N27	0.0133 (5)	0.0186 (5)	0.0119 (5)	−0.0020 (4)	0.0018 (4)	−0.0004 (4)
C28	0.0166 (6)	0.0202 (6)	0.0165 (6)	−0.0022 (5)	0.0070 (5)	0.0002 (5)
O28	0.0298 (5)	0.0182 (5)	0.0266 (5)	−0.0019 (4)	0.0068 (4)	0.0005 (4)
C29	0.0180 (6)	0.0265 (7)	0.0263 (7)	−0.0004 (5)	0.0067 (5)	−0.0024 (6)
O29	0.0315 (6)	0.0279 (6)	0.0356 (6)	−0.0008 (5)	0.0003 (5)	−0.0092 (5)
C30	0.0249 (7)	0.0306 (8)	0.0349 (8)	0.0064 (6)	0.0029 (6)	−0.0010 (7)
C31	0.0155 (6)	0.0165 (6)	0.0121 (5)	−0.0008 (5)	0.0006 (4)	0.0014 (4)
C32	0.0166 (6)	0.0171 (6)	0.0147 (6)	0.0005 (5)	0.0016 (5)	0.0000 (5)
C33	0.0192 (6)	0.0182 (6)	0.0137 (6)	0.0004 (5)	0.0034 (5)	−0.0027 (5)
O33	0.0178 (5)	0.0355 (6)	0.0245 (5)	0.0042 (4)	0.0042 (4)	0.0056 (4)
O34	0.0209 (5)	0.0289 (5)	0.0231 (5)	0.0031 (4)	0.0082 (4)	0.0099 (4)
C34	0.0299 (8)	0.0269 (8)	0.0300 (8)	0.0000 (6)	0.0156 (6)	0.0087 (6)
C35	0.0192 (6)	0.0186 (6)	0.0137 (6)	0.0010 (5)	0.0028 (5)	0.0007 (5)
O35	0.0238 (5)	0.0200 (5)	0.0355 (6)	−0.0045 (4)	0.0105 (4)	−0.0014 (4)
O36	0.0199 (5)	0.0163 (5)	0.0266 (5)	0.0025 (4)	0.0044 (4)	−0.0001 (4)
C36	0.0338 (8)	0.0174 (7)	0.0292 (8)	0.0052 (6)	0.0069 (6)	−0.0006 (6)

### Geometric parameters (Å, º)

**Table:**

C1—N27	1.4862 (16)	C20—C21	1.5143 (17)
C1—C2	1.5124 (17)	C21—N27	1.4854 (16)
C1—C26	1.5560 (18)	C21—C22	1.5548 (17)
C1—H1	1.0000	C21—H21	1.0000
C2—C3	1.3907 (19)	C22—C28	1.5081 (18)
C2—C7	1.4025 (19)	C22—C23	1.5456 (18)
C3—C4	1.3962 (19)	C22—H22	1.0000
C3—H3	0.9500	C23—N24	1.4576 (17)
C4—C5	1.380 (2)	C23—H23A	0.9900
C4—H4	0.9500	C23—H23B	0.9900
C5—C6	1.386 (2)	N24—C29	1.3623 (19)
C5—H5	0.9500	N24—C25	1.4621 (17)
C6—C7	1.3944 (19)	C25—C26	1.5435 (18)
C6—H6	0.9500	C25—H25A	0.9900
C7—O8	1.3603 (17)	C25—H25B	0.9900
O8—C9	1.4338 (17)	C26—C28	1.5097 (18)
C9—C10	1.500 (2)	C26—H26	1.0000
C9—H9A	0.9900	N27—C31	1.4245 (16)
C9—H9B	0.9900	C28—O28	1.2130 (16)
C10—O11	1.4228 (17)	C29—O29	1.2283 (18)
C10—H10A	0.9900	C29—C30	1.509 (2)
C10—H10B	0.9900	C30—H30A	0.9800
O11—C12	1.4196 (17)	C30—H30B	0.9800
C12—C13	1.500 (2)	C30—H30C	0.9800
C12—H12A	0.9900	C31—C32	1.3311 (18)
C12—H12B	0.9900	C31—C35	1.5036 (18)
C13—O14	1.4292 (16)	C32—C33	1.4799 (18)
C13—H13A	0.9900	C32—H32	0.9500
C13—H13B	0.9900	C33—O33	1.2076 (16)
O14—C15	1.3650 (15)	C33—O34	1.3337 (16)
C15—C16	1.3950 (18)	O34—C34	1.4512 (16)
C15—C20	1.4086 (18)	C34—H34A	0.9800
C16—C17	1.3894 (19)	C34—H34B	0.9800
C16—H16	0.9500	C34—H34C	0.9800
C17—C18	1.3804 (19)	C35—O35	1.2019 (16)
C17—H17	0.9500	C35—O36	1.3373 (16)
C18—C19	1.3942 (18)	O36—C36	1.4481 (17)
C18—H18	0.9500	C36—H36A	0.9800
C19—C20	1.3885 (18)	C36—H36B	0.9800
C19—H19	0.9500	C36—H36C	0.9800
			
N27—C1—C2	114.34 (10)	N27—C21—H21	108.1
N27—C1—C26	107.57 (10)	C20—C21—H21	108.1
C2—C1—C26	111.62 (10)	C22—C21—H21	108.1
N27—C1—H1	107.7	C28—C22—C23	105.85 (10)
C2—C1—H1	107.7	C28—C22—C21	110.33 (10)
C26—C1—H1	107.7	C23—C22—C21	110.96 (11)
C3—C2—C7	118.54 (12)	C28—C22—H22	109.9
C3—C2—C1	119.31 (12)	C23—C22—H22	109.9
C7—C2—C1	122.01 (12)	C21—C22—H22	109.9
C2—C3—C4	121.13 (14)	N24—C23—C22	110.07 (10)
C2—C3—H3	119.4	N24—C23—H23A	109.6
C4—C3—H3	119.4	C22—C23—H23A	109.6
C5—C4—C3	119.31 (14)	N24—C23—H23B	109.6
C5—C4—H4	120.3	C22—C23—H23B	109.6
C3—C4—H4	120.3	H23A—C23—H23B	108.2
C4—C5—C6	120.89 (13)	C29—N24—C23	120.37 (11)
C4—C5—H5	119.6	C29—N24—C25	125.43 (12)
C6—C5—H5	119.6	C23—N24—C25	114.19 (11)
C5—C6—C7	119.59 (14)	N24—C25—C26	110.62 (10)
C5—C6—H6	120.2	N24—C25—H25A	109.5
C7—C6—H6	120.2	C26—C25—H25A	109.5
O8—C7—C6	123.83 (13)	N24—C25—H25B	109.5
O8—C7—C2	115.64 (11)	C26—C25—H25B	109.5
C6—C7—C2	120.50 (13)	H25A—C25—H25B	108.1
C7—O8—C9	119.25 (11)	C28—C26—C25	105.72 (10)
O8—C9—C10	105.72 (11)	C28—C26—C1	110.66 (10)
O8—C9—H9A	110.6	C25—C26—C1	111.13 (11)
C10—C9—H9A	110.6	C28—C26—H26	109.8
O8—C9—H9B	110.6	C25—C26—H26	109.8
C10—C9—H9B	110.6	C1—C26—H26	109.8
H9A—C9—H9B	108.7	C31—N27—C21	113.99 (10)
O11—C10—C9	107.99 (12)	C31—N27—C1	116.30 (10)
O11—C10—H10A	110.1	C21—N27—C1	110.20 (9)
C9—C10—H10A	110.1	O28—C28—C22	124.07 (12)
O11—C10—H10B	110.1	O28—C28—C26	124.59 (12)
C9—C10—H10B	110.1	C22—C28—C26	110.61 (11)
H10A—C10—H10B	108.4	O29—C29—N24	121.24 (13)
C12—O11—C10	112.57 (11)	O29—C29—C30	120.99 (14)
O11—C12—C13	109.13 (12)	N24—C29—C30	117.76 (13)
O11—C12—H12A	109.9	C29—C30—H30A	109.5
C13—C12—H12A	109.9	C29—C30—H30B	109.5
O11—C12—H12B	109.9	H30A—C30—H30B	109.5
C13—C12—H12B	109.9	C29—C30—H30C	109.5
H12A—C12—H12B	108.3	H30A—C30—H30C	109.5
O14—C13—C12	107.14 (11)	H30B—C30—H30C	109.5
O14—C13—H13A	110.3	C32—C31—N27	119.05 (12)
C12—C13—H13A	110.3	C32—C31—C35	121.12 (12)
O14—C13—H13B	110.3	N27—C31—C35	119.75 (11)
C12—C13—H13B	110.3	C31—C32—C33	126.45 (12)
H13A—C13—H13B	108.5	C31—C32—H32	116.8
C15—O14—C13	117.64 (10)	C33—C32—H32	116.8
O14—C15—C16	123.71 (12)	O33—C33—O34	123.74 (12)
O14—C15—C20	115.90 (11)	O33—C33—C32	121.90 (12)
C16—C15—C20	120.33 (12)	O34—C33—C32	114.26 (11)
C17—C16—C15	119.87 (12)	C33—O34—C34	114.68 (11)
C17—C16—H16	120.1	O34—C34—H34A	109.5
C15—C16—H16	120.1	O34—C34—H34B	109.5
C18—C17—C16	120.73 (12)	H34A—C34—H34B	109.5
C18—C17—H17	119.6	O34—C34—H34C	109.5
C16—C17—H17	119.6	H34A—C34—H34C	109.5
C17—C18—C19	119.04 (12)	H34B—C34—H34C	109.5
C17—C18—H18	120.5	O35—C35—O36	124.79 (12)
C19—C18—H18	120.5	O35—C35—C31	124.67 (12)
C20—C19—C18	121.91 (12)	O36—C35—C31	110.53 (11)
C20—C19—H19	119.0	C35—O36—C36	117.33 (11)
C18—C19—H19	119.0	O36—C36—H36A	109.5
C19—C20—C15	118.10 (11)	O36—C36—H36B	109.5
C19—C20—C21	119.71 (11)	H36A—C36—H36B	109.5
C15—C20—C21	122.07 (11)	O36—C36—H36C	109.5
N27—C21—C20	113.17 (10)	H36A—C36—H36C	109.5
N27—C21—C22	107.93 (10)	H36B—C36—H36C	109.5
C20—C21—C22	111.15 (10)		
			
N27—C1—C2—C3	−118.70 (13)	C22—C23—N24—C29	−122.32 (13)
C26—C1—C2—C3	118.90 (13)	C22—C23—N24—C25	56.46 (14)
N27—C1—C2—C7	65.64 (16)	C29—N24—C25—C26	122.45 (14)
C26—C1—C2—C7	−56.75 (16)	C23—N24—C25—C26	−56.26 (15)
C7—C2—C3—C4	1.7 (2)	N24—C25—C26—C28	57.46 (14)
C1—C2—C3—C4	−174.11 (12)	N24—C25—C26—C1	−62.65 (14)
C2—C3—C4—C5	−0.1 (2)	N27—C1—C26—C28	2.43 (14)
C3—C4—C5—C6	−1.5 (2)	C2—C1—C26—C28	128.63 (11)
C4—C5—C6—C7	1.3 (2)	N27—C1—C26—C25	119.57 (11)
C5—C6—C7—O8	178.27 (13)	C2—C1—C26—C25	−114.23 (12)
C5—C6—C7—C2	0.3 (2)	C20—C21—N27—C31	−38.88 (14)
C3—C2—C7—O8	−179.92 (12)	C22—C21—N27—C31	−162.31 (10)
C1—C2—C7—O8	−4.24 (18)	C20—C21—N27—C1	−171.75 (10)
C3—C2—C7—C6	−1.8 (2)	C22—C21—N27—C1	64.81 (12)
C1—C2—C7—C6	173.87 (12)	C2—C1—N27—C31	39.86 (16)
C6—C7—O8—C9	12.0 (2)	C26—C1—N27—C31	164.43 (10)
C2—C7—O8—C9	−169.97 (12)	C2—C1—N27—C21	171.54 (11)
C7—O8—C9—C10	169.85 (11)	C26—C1—N27—C21	−63.88 (13)
O8—C9—C10—O11	−67.15 (14)	C23—C22—C28—O28	−105.51 (14)
C9—C10—O11—C12	164.25 (12)	C21—C22—C28—O28	134.37 (13)
C10—O11—C12—C13	−156.42 (12)	C23—C22—C28—C26	65.06 (13)
O11—C12—C13—O14	70.09 (14)	C21—C22—C28—C26	−55.06 (13)
C12—C13—O14—C15	−179.32 (11)	C25—C26—C28—O28	106.01 (14)
C13—O14—C15—C16	−17.86 (18)	C1—C26—C28—O28	−133.57 (13)
C13—O14—C15—C20	164.85 (11)	C25—C26—C28—C22	−64.49 (13)
O14—C15—C16—C17	−176.19 (12)	C1—C26—C28—C22	55.92 (13)
C20—C15—C16—C17	0.99 (19)	C23—N24—C29—O29	−0.9 (2)
C15—C16—C17—C18	−0.8 (2)	C25—N24—C29—O29	−179.56 (13)
C16—C17—C18—C19	0.0 (2)	C23—N24—C29—C30	179.47 (12)
C17—C18—C19—C20	0.7 (2)	C25—N24—C29—C30	0.8 (2)
C18—C19—C20—C15	−0.51 (19)	C21—N27—C31—C32	127.63 (12)
C18—C19—C20—C21	175.64 (12)	C1—N27—C31—C32	−102.47 (14)
O14—C15—C20—C19	177.05 (11)	C21—N27—C31—C35	−49.22 (15)
C16—C15—C20—C19	−0.34 (18)	C1—N27—C31—C35	80.68 (14)
O14—C15—C20—C21	0.99 (17)	N27—C31—C32—C33	−175.87 (11)
C16—C15—C20—C21	−176.40 (11)	C35—C31—C32—C33	0.9 (2)
C19—C20—C21—N27	126.37 (12)	C31—C32—C33—O33	119.73 (16)
C15—C20—C21—N27	−57.63 (15)	C31—C32—C33—O34	−63.77 (18)
C19—C20—C21—C22	−111.98 (13)	O33—C33—O34—C34	−5.65 (19)
C15—C20—C21—C22	64.01 (15)	C32—C33—O34—C34	177.93 (12)
N27—C21—C22—C28	−3.72 (14)	C32—C31—C35—O35	−28.2 (2)
C20—C21—C22—C28	−128.38 (11)	N27—C31—C35—O35	148.54 (13)
N27—C21—C22—C23	−120.71 (11)	C32—C31—C35—O36	152.95 (12)
C20—C21—C22—C23	114.63 (11)	N27—C31—C35—O36	−30.27 (15)
C28—C22—C23—N24	−58.32 (14)	O35—C35—O36—C36	−2.02 (19)
C21—C22—C23—N24	61.39 (13)	C31—C35—O36—C36	176.78 (11)

### Hydrogen-bond geometry (Å, º)

**Table:**

*D*—H···*A*	*D*—H	H···*A*	*D*···*A*	*D*—H···*A*
C18—H18···O35^i^	0.95	2.47	3.1735 (17)	131
C25—H25*A*···O33^ii^	0.99	2.30	3.2091 (17)	152
C34—H34*A*···O35^iii^	0.98	2.53	3.5045 (19)	174

Symmetry codes: (i) *x*, −*y*+1/2, *z*+1/2; (ii) *x*−1, *y*, *z*; (iii) *x*, −*y*+1/2, *z*−1/2.
